# *catena*-Poly[[[tetraaqua(3,5-dinitro-4-oxidopyridine *N*-oxide-κ*O* ^1^)neodymium(III)]-μ-oxalato-κ^4^ *O* ^1^,*O* ^2^:*O* ^1′^,*O* ^2′^] tetrahydrate]

**DOI:** 10.1107/S1600536810041139

**Published:** 2010-10-23

**Authors:** Yin-Feng Han, Seik Weng Ng

**Affiliations:** a Department of Chemistry and Chemical Engineering, Baoji University of Arts and Science, Baoji 721013, People’s Republic of China; b Department of Chemistry, University of Malaya, 50603 Kuala Lumpur, Malaysia

## Abstract

In the title coordination polymer, {[Nd(C_5_H_2_N_3_O_6_)(C_2_O_4_)(H_2_O)_4_]·4H_2_O}_*n*_, the oxalate dianions link adjacent nine-coordinate, tricapped trigonal-prismatic Nd(III) atoms into a chain running along the *b* axis. The 3,5-dinitropyridin-4-oxido *N*-oxide ligand is formally a zwitterionic anion; the anion binds to the metal atom through the *N*-oxide O atom. The chains are connected into a three-dimensional network by O—H⋯O hydrogen bonds involving the coordinated and uncoordinated water mol­ecules.

## Related literature

For a related Nd(III) structure, see: Wang *et al.* (2010 ▶).
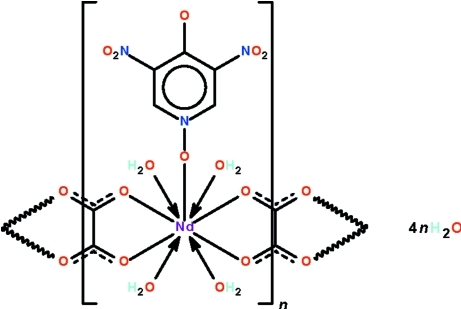


## Experimental

### 

#### Crystal data

[Nd(C_5_H_2_N_3_O_6_)(C_2_O_4_)(H_2_O)_4_]·4H_2_O
*M* *_r_* = 576.48
Triclinic, 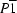

*a* = 6.7695 (7) Å
*b* = 9.9695 (11) Å
*c* = 14.6269 (16) Å
α = 73.719 (1)°
β = 88.137 (1)°
γ = 76.693 (1)°
*V* = 921.58 (17) Å^3^
*Z* = 2
Mo *K*α radiation
μ = 2.92 mm^−1^
*T* = 293 K
0.35 × 0.25 × 0.05 mm

#### Data collection

Bruker SMART APEX diffractometer
Absorption correction: multi-scan (*SADABS*; Sheldrick, 1996 ▶) *T*_min_ = 0.429, *T*_max_ = 0.868
7952 measured reflections
4147 independent reflections
4005 reflections with *I* > 2σ(*I*)
*R*_int_ = 0.015

#### Refinement

*R*[*F* ^2^ > 2σ(*F* ^2^)] = 0.019
*wR*(*F* ^2^) = 0.050
*S* = 1.06
4147 reflections
262 parameters
H-atom parameters constrained
Δρ_max_ = 0.48 e Å^−3^
Δρ_min_ = −0.94 e Å^−3^

### 

Data collection: *SMART* (Bruker, 2003 ▶); cell refinement: *SAINT* (Bruker, 2003 ▶); data reduction: *SAINT*; program(s) used to solve structure: *SHELXS97* (Sheldrick, 2008 ▶); program(s) used to refine structure: *SHELXL97* (Sheldrick, 2008 ▶); molecular graphics: *X-SEED* (Barbour, 2001 ▶); software used to prepare material for publication: *publCIF* (Westrip, 2010 ▶).

## Supplementary Material

Crystal structure: contains datablocks global, I. DOI: 10.1107/S1600536810041139/xu5029sup1.cif

Structure factors: contains datablocks I. DOI: 10.1107/S1600536810041139/xu5029Isup2.hkl

Additional supplementary materials:  crystallographic information; 3D view; checkCIF report

## Figures and Tables

**Table 1:** Selected bond lengths (Å)

Nd1—O1	2.5266 (16)
Nd1—O7	2.5124 (16)
Nd1—O8^i^	2.4972 (16)
Nd1—O9	2.5243 (16)
Nd1—O10^ii^	2.4897 (16)
Nd1—O1*W*	2.4798 (16)
Nd1—O2*W*	2.5080 (17)
Nd1—O3*W*	2.5241 (17)
Nd1—O4*W*	2.5153 (16)

Symmetry codes: (i) 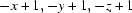; (ii) 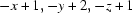.

**Table 2:** Hydrogen-bond geometry (Å, °)

*D*—H⋯*A*	*D*—H	H⋯*A*	*D*⋯*A*	*D*—H⋯*A*
O1w—H11⋯O4^iii^	0.84	1.99	2.809 (2)	166
O1w—H12⋯O5w	0.84	1.89	2.736 (3)	178
O2w—H21⋯O8^iv^	0.84	2.06	2.890 (2)	174
O2w—H22⋯O7w	0.84	1.97	2.806 (3)	174
O3w—H31⋯O7^iv^	0.85	2.07	2.908 (2)	170
O3w—H32⋯O8w^iv^	0.84	2.03	2.841 (3)	161
O4w—H41⋯O4^iii^	0.84	1.97	2.759 (2)	155
O4w—H42⋯O6w^v^	0.83	2.02	2.851 (3)	177
O5w—H51⋯O6w	0.84	2.06	2.893 (3)	171
O5w—H52⋯O3^iii^	0.82	2.43	2.956 (3)	123
O6w—H61⋯O10	0.84	2.09	2.915 (3)	168
O6w—H62⋯O8w^ii^	0.85	2.13	2.942 (3)	160
O7w—H71⋯O1^ii^	0.84	1.93	2.766 (2)	172
O7w—H72⋯O9^vi^	0.84	2.13	2.953 (2)	168
O8w—H81⋯O6^ii^	0.85	2.46	3.310 (4)	174
O8w—H82⋯O7w	0.84	2.02	2.816 (3)	160

Symmetry codes: (ii) 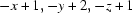; (iii) 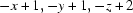; (iv) 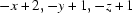; (v) 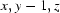; (vi) 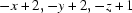.

**Fig. 1.**
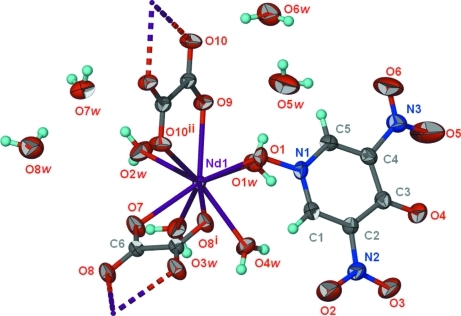
Thermal ellipsoid plot (Barbour, 2001) of a portion of the chain motif of [Nd(H2O)4(C5H2N3O6)(C2O4).H2O]n at the 50% probability level; hydrogen atoms are shown as spheres of arbitrary radius. Symmetry codes: i = 1 - x, 1 - y, 1 - z; ii = 1 - y, 2 - y, 1 - z.

**Fig. 2.**
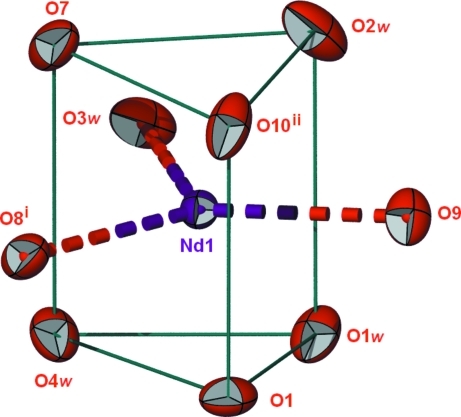
Nine-coordinate geometry of Nd.

## supplementary crystallographic information

### Comment

Lanthanum(III) oxalates adopt open-framework architectures whose cavities are occupied by the templating agent, typically an ammonium counterion. The reaction of neodymium nitrate and adipic acid in the presence of 3,5-dinitro-4-hydroxypyridine *N*-oxide gave an unexpected neodymium oxalate, whose positive charge is balanced by the deprotonated 3,5-dinitropyridin-4-olate-*N*-oxide unit. The oxalate part of the coordination polymer, [Nd(H_2_O)_4_(C_5_H_2_N_3_O_6_)(C_2_O_4_)^.^H_2_O]*_n_* (Scheme I, Fig. 1), links adjacent nine-coordinate, tricapped-trigonal-prismatic Nd(III) centers (Fig. 2) into a linear chain running along the *b* axis of the triclinic unit cell. The 3,5-dinitropyridin-4-olate-*N*-oxide part is formally a zwitterionic anion; the anion binds through the *N*-oxide O atom. The chains are connected by O–H···O hydrogen bonds that involve the coordinated and lattice water molecules into a three-dimensional network.

This study continues from the only report of a metal derivative of 3,5-dinitro-4-hydroxypyridine *N*-oxide; the deprotonated ligand is also unidentate in the tris-ethanol adduct of the same metal (Wang *et al.*, 2010).

### Experimental

3,5-Dinitro-4-hydroxypyridine *N*-oxide (0.183 g), a commercially available chemical, was dissolved in water (25 ml) at 333 K. Neodymium nitrate hexahydrate (0.441 g) was added and the mixture heated for 6 h. Adipic acid (0.091 g) was added and the mixture heated for another 2 h. The solution was filtered and the water removed by evaporation. The residue was recrystallzed from ethanol solution to furnish violet-colored prisms. These were washed with water.

### Refinement

Carbon-bound H-atoms were placed in calculated positions (C–H 0.93 Å) and were included in the refinement in the riding model approximation, with *U*(H) set to 1.2*U*(C).

The water H atoms were placed in chemically sensible positions on the basis of hydrogen bonding (O–H 0.82–0.85 Å) and their temperature factors tied by a factor of 1.5 times.

### Crystal data

**Table:**

[Nd(C_5_H_2_N_3_O_6_)(C_2_O_4_)(H_2_O)_4_]·4H_2_O	*Z* = 2
*M**_r_* = 576.48	*F*(000) = 570
Triclinic, *P*1	*D*_x_ = 2.077 Mg m^−^^3^
Hall symbol: -P 1	Mo *K*α radiation, λ = 0.71073 Å
*a* = 6.7695 (7) Å	Cell parameters from 6995 reflections
*b* = 9.9695 (11) Å	θ = 2.3–28.2°
*c* = 14.6269 (16) Å	µ = 2.92 mm^−^^1^
α = 73.719 (1)°	*T* = 293 K
β = 88.137 (1)°	Prism, violet
γ = 76.693 (1)°	0.35 × 0.25 × 0.05 mm
*V* = 921.58 (17) Å^3^	

### Data collection

**Table:**

Bruker SMART APEX diffraractometer diffractometer	4147 independent reflections
Radiation source: fine-focus sealed tube	4005 reflections with *I* > 2σ(*I*)
graphite	*R*_int_ = 0.015
φ and ω scans	θ_max_ = 27.5°, θ_min_ = 2.3°
Absorption correction: multi-scan (*SADABS*; Sheldrick, 1996)	*h* = −8→8
*T*_min_ = 0.429, *T*_max_ = 0.868	*k* = −12→12
7952 measured reflections	*l* = −18→18

### Refinement

**Table:**

Refinement on *F*^2^	Primary atom site location: structure-invariant direct methods
Least-squares matrix: full	Secondary atom site location: difference Fourier map
*R*[*F*^2^ > 2σ(*F*^2^)] = 0.019	Hydrogen site location: inferred from neighbouring sites
*wR*(*F*^2^) = 0.050	H-atom parameters constrained
*S* = 1.06	*w* = 1/[σ^2^(*F*_o_^2^) + (0.031*P*)^2^ + 0.4577*P*] where *P* = (*F*_o_^2^ + 2*F*_c_^2^)/3
4147 reflections	(Δ/σ)_max_ = 0.001
262 parameters	Δρ_max_ = 0.48 e Å^−^^3^
0 restraints	Δρ_min_ = −0.94 e Å^−^^3^

### Fractional atomic coordinates and isotropic or equivalent isotropic displacement parameters (Å^2^)

**Table:**

	*x*	*y*	*z*	*U*_iso_*/*U*_eq_	
Nd1	0.671311 (14)	0.671999 (10)	0.625076 (7)	0.01518 (5)	
N1	0.3029 (3)	0.7237 (2)	0.78523 (13)	0.0226 (4)	
N2	0.2172 (3)	0.3827 (2)	0.93382 (15)	0.0299 (4)	
N3	0.2733 (4)	0.8438 (2)	1.00008 (16)	0.0403 (6)	
O1	0.3422 (3)	0.77791 (18)	0.69178 (11)	0.0261 (3)	
O1W	0.7817 (3)	0.69793 (18)	0.77798 (11)	0.0292 (4)	
H11	0.7867	0.6312	0.8283	0.044*	
H12	0.7757	0.7744	0.7930	0.044*	
O2	0.2029 (5)	0.3315 (3)	0.86815 (16)	0.0645 (8)	
O2W	0.9719 (3)	0.75337 (19)	0.54317 (15)	0.0383 (4)	
H21	1.0937	0.7091	0.5550	0.057*	
H22	0.9657	0.8251	0.4958	0.057*	
O3	0.2085 (4)	0.3157 (2)	1.01719 (14)	0.0471 (5)	
O3W	1.0000 (3)	0.48281 (19)	0.66002 (13)	0.0340 (4)	
H31	1.0649	0.4802	0.6098	0.051*	
H32	1.0039	0.3978	0.6927	0.051*	
O4	0.2421 (3)	0.5451 (2)	1.07188 (12)	0.0361 (4)	
O4W	0.6382 (3)	0.44826 (18)	0.75126 (12)	0.0284 (4)	
H41	0.6831	0.4230	0.8078	0.043*	
H42	0.6051	0.3790	0.7399	0.043*	
O5	0.2961 (8)	0.7979 (3)	1.08425 (18)	0.1165 (17)	
O5W	0.7576 (5)	0.9490 (3)	0.82384 (19)	0.0745 (9)	
H51	0.6753	1.0248	0.7945	0.112*	
H52	0.7493	0.9288	0.8820	0.112*	
O6	0.2434 (7)	0.9714 (3)	0.96151 (19)	0.0894 (12)	
O6W	0.5130 (3)	1.2177 (2)	0.70856 (15)	0.0448 (5)	
H61	0.5106	1.2141	0.6518	0.067*	
H62	0.4016	1.2031	0.7331	0.067*	
O7	0.7357 (2)	0.5438 (2)	0.49783 (13)	0.0303 (4)	
O7W	0.9661 (3)	0.98025 (19)	0.37762 (14)	0.0361 (4)	
H71	0.8719	1.0540	0.3619	0.054*	
H72	1.0779	1.0030	0.3739	0.054*	
O8	0.6186 (2)	0.41980 (19)	0.41502 (13)	0.0270 (3)	
O8W	0.9041 (4)	0.8190 (2)	0.25538 (16)	0.0513 (6)	
H81	0.8735	0.8759	0.1999	0.077*	
H82	0.9535	0.8569	0.2908	0.077*	
O9	0.6495 (2)	0.93475 (16)	0.60571 (11)	0.0248 (3)	
O10	0.5087 (3)	1.16112 (17)	0.52372 (12)	0.0306 (4)	
C1	0.2625 (3)	0.5922 (2)	0.81517 (16)	0.0227 (4)	
H1	0.2476	0.5435	0.7710	0.027*	
C2	0.2433 (3)	0.5296 (2)	0.91041 (16)	0.0222 (4)	
C3	0.2536 (3)	0.6000 (2)	0.98472 (16)	0.0236 (4)	
C4	0.2774 (4)	0.7458 (2)	0.94198 (16)	0.0260 (5)	
C5	0.3056 (4)	0.8018 (2)	0.84657 (17)	0.0260 (5)	
H5	0.3266	0.8943	0.8243	0.031*	
C6	0.6016 (3)	0.4897 (2)	0.47487 (15)	0.0197 (4)	
C7	0.5458 (3)	1.0272 (2)	0.53784 (15)	0.0209 (4)	

### Atomic displacement parameters (Å^2^)

**Table:**

	*U*^11^	*U*^22^	*U*^33^	*U*^12^	*U*^13^	*U*^23^
Nd1	0.01768 (7)	0.01469 (7)	0.01310 (6)	−0.00390 (4)	−0.00032 (4)	−0.00356 (4)
N1	0.0235 (9)	0.0255 (9)	0.0170 (8)	−0.0038 (7)	0.0025 (7)	−0.0049 (7)
N2	0.0405 (11)	0.0271 (10)	0.0236 (10)	−0.0120 (9)	0.0007 (8)	−0.0063 (8)
N3	0.0708 (17)	0.0278 (11)	0.0241 (11)	−0.0114 (11)	−0.0001 (11)	−0.0096 (9)
O1	0.0309 (8)	0.0277 (8)	0.0163 (7)	−0.0035 (7)	0.0063 (6)	−0.0041 (6)
O1W	0.0431 (10)	0.0254 (8)	0.0193 (8)	−0.0061 (7)	−0.0061 (7)	−0.0071 (6)
O2	0.131 (3)	0.0454 (13)	0.0338 (11)	−0.0453 (15)	0.0084 (13)	−0.0179 (10)
O2W	0.0260 (8)	0.0279 (9)	0.0506 (12)	−0.0042 (7)	0.0098 (8)	0.0028 (8)
O3	0.0825 (16)	0.0360 (11)	0.0251 (9)	−0.0260 (11)	0.0002 (10)	−0.0019 (8)
O3W	0.0295 (9)	0.0278 (9)	0.0340 (9)	0.0009 (7)	0.0082 (7)	0.0015 (7)
O4	0.0581 (12)	0.0327 (10)	0.0181 (8)	−0.0146 (9)	−0.0014 (8)	−0.0045 (7)
O4W	0.0374 (9)	0.0250 (8)	0.0223 (8)	−0.0126 (7)	−0.0011 (7)	−0.0011 (6)
O5	0.289 (6)	0.0440 (15)	0.0247 (12)	−0.051 (2)	−0.018 (2)	−0.0095 (10)
O5W	0.143 (3)	0.0332 (12)	0.0434 (14)	−0.0063 (15)	−0.0147 (16)	−0.0135 (10)
O6	0.196 (4)	0.0310 (13)	0.0439 (14)	−0.0246 (17)	0.0042 (19)	−0.0161 (11)
O6W	0.0596 (13)	0.0429 (11)	0.0366 (11)	−0.0140 (10)	−0.0045 (10)	−0.0163 (9)
O7	0.0230 (8)	0.0416 (10)	0.0379 (10)	−0.0121 (7)	0.0062 (7)	−0.0264 (8)
O7W	0.0364 (10)	0.0282 (9)	0.0418 (10)	−0.0110 (7)	−0.0046 (8)	−0.0035 (8)
O8	0.0240 (8)	0.0359 (9)	0.0300 (9)	−0.0120 (7)	0.0066 (7)	−0.0200 (7)
O8W	0.0771 (16)	0.0401 (12)	0.0403 (12)	−0.0189 (11)	−0.0021 (11)	−0.0123 (9)
O9	0.0309 (8)	0.0195 (7)	0.0222 (8)	−0.0056 (6)	−0.0070 (6)	−0.0022 (6)
O10	0.0488 (10)	0.0176 (8)	0.0240 (8)	−0.0041 (7)	−0.0123 (7)	−0.0049 (6)
C1	0.0215 (10)	0.0253 (11)	0.0215 (10)	−0.0038 (8)	0.0016 (8)	−0.0083 (8)
C2	0.0223 (10)	0.0236 (11)	0.0207 (10)	−0.0055 (8)	0.0008 (8)	−0.0061 (8)
C3	0.0250 (10)	0.0254 (11)	0.0198 (10)	−0.0041 (8)	−0.0006 (8)	−0.0067 (8)
C4	0.0333 (12)	0.0242 (11)	0.0218 (11)	−0.0055 (9)	0.0002 (9)	−0.0095 (9)
C5	0.0312 (11)	0.0216 (11)	0.0256 (11)	−0.0062 (9)	0.0028 (9)	−0.0071 (9)
C6	0.0191 (10)	0.0205 (10)	0.0202 (10)	−0.0034 (8)	0.0015 (8)	−0.0078 (8)
C7	0.0238 (10)	0.0197 (10)	0.0187 (10)	−0.0053 (8)	−0.0011 (8)	−0.0043 (8)

### Geometric parameters (Å, °)

**Table:**

Nd1—O1	2.5266 (16)	O4—C3	1.246 (3)
Nd1—O7	2.5124 (16)	O4W—H41	0.8391
Nd1—O8^i^	2.4972 (16)	O4W—H42	0.8323
Nd1—O9	2.5243 (16)	O5W—H51	0.8401
Nd1—O10^ii^	2.4897 (16)	O5W—H52	0.8205
Nd1—O1W	2.4798 (16)	O6W—H61	0.8420
Nd1—O2W	2.5080 (17)	O6W—H62	0.8503
Nd1—O3W	2.5241 (17)	O7—C6	1.255 (3)
Nd1—O4W	2.5153 (16)	O7W—H71	0.8363
N1—C5	1.346 (3)	O7W—H72	0.8351
N1—C1	1.350 (3)	O8—C6	1.251 (3)
N1—O1	1.364 (2)	O8W—H81	0.8498
N2—O2	1.223 (3)	O8W—H82	0.8376
N2—O3	1.222 (3)	O9—C7	1.250 (3)
N2—C2	1.459 (3)	O10—C7	1.258 (3)
N3—O5	1.189 (3)	C1—C2	1.373 (3)
N3—O6	1.210 (3)	C1—H1	0.9300
N3—C4	1.459 (3)	C2—C3	1.461 (3)
O1W—H11	0.8381	C3—C4	1.456 (3)
O1W—H12	0.8435	C4—C5	1.375 (3)
O2W—H21	0.8386	C5—H5	0.9300
O2W—H22	0.8392	C6—C6^i^	1.538 (4)
O3W—H31	0.8474	C7—C7^ii^	1.563 (4)
O3W—H32	0.8431		
			
O1W—Nd1—O10^ii^	135.33 (5)	O6—N3—C4	119.1 (2)
O1W—Nd1—O8^i^	131.00 (6)	N1—O1—Nd1	119.90 (12)
O10^ii^—Nd1—O8^i^	70.29 (6)	Nd1—O1W—H11	120.2
O1W—Nd1—O2W	91.36 (6)	Nd1—O1W—H12	127.9
O10^ii^—Nd1—O2W	81.90 (6)	H11—O1W—H12	108.0
O8^i^—Nd1—O2W	137.49 (6)	Nd1—O2W—H21	125.7
O1W—Nd1—O7	148.48 (6)	Nd1—O2W—H22	125.1
O10^ii^—Nd1—O7	72.57 (6)	H21—O2W—H22	108.9
O8^i^—Nd1—O7	64.62 (5)	Nd1—O3W—H31	111.6
O2W—Nd1—O7	76.89 (6)	Nd1—O3W—H32	121.7
O1W—Nd1—O4W	73.70 (6)	H31—O3W—H32	108.4
O10^ii^—Nd1—O4W	139.35 (6)	Nd1—O4W—H41	126.0
O8^i^—Nd1—O4W	69.18 (6)	Nd1—O4W—H42	123.9
O2W—Nd1—O4W	132.51 (6)	H41—O4W—H42	108.6
O7—Nd1—O4W	92.70 (6)	H51—O5W—H52	114.0
O1W—Nd1—O9	71.57 (5)	H61—O6W—H62	108.1
O10^ii^—Nd1—O9	65.01 (5)	C6—O7—Nd1	120.14 (14)
O8^i^—Nd1—O9	122.97 (6)	H71—O7W—H72	110.0
O2W—Nd1—O9	67.67 (6)	C6—O8—Nd1^i^	121.03 (14)
O7—Nd1—O9	127.25 (6)	H81—O8W—H82	111.9
O4W—Nd1—O9	140.03 (6)	C7—O9—Nd1	119.52 (14)
O1W—Nd1—O3W	79.37 (6)	C7—O10—Nd1^ii^	121.18 (14)
O10^ii^—Nd1—O3W	134.04 (6)	N1—C1—C2	120.4 (2)
O8^i^—Nd1—O3W	112.90 (6)	N1—C1—H1	119.8
O2W—Nd1—O3W	65.61 (6)	C2—C1—H1	119.8
O7—Nd1—O3W	69.13 (6)	C1—C2—N2	114.8 (2)
O4W—Nd1—O3W	67.38 (6)	C1—C2—C3	123.9 (2)
O9—Nd1—O3W	123.39 (6)	N2—C2—C3	121.22 (19)
O1W—Nd1—O1	76.77 (6)	O4—C3—C4	124.5 (2)
O10^ii^—Nd1—O1	79.67 (6)	O4—C3—C2	125.6 (2)
O8^i^—Nd1—O1	67.82 (6)	C4—C3—C2	109.93 (19)
O2W—Nd1—O1	138.53 (6)	C5—C4—N3	115.0 (2)
O7—Nd1—O1	130.56 (5)	C5—C4—C3	124.1 (2)
O4W—Nd1—O1	82.50 (6)	N3—C4—C3	120.9 (2)
O9—Nd1—O1	70.89 (5)	N1—C5—C4	120.4 (2)
O3W—Nd1—O1	145.78 (5)	N1—C5—H5	119.8
C5—N1—C1	120.8 (2)	C4—C5—H5	119.8
C5—N1—O1	119.60 (19)	O8—C6—O7	125.9 (2)
C1—N1—O1	119.60 (18)	O8—C6—C6^i^	116.9 (2)
O2—N2—O3	122.3 (2)	O7—C6—C6^i^	117.2 (2)
O2—N2—C2	118.1 (2)	O9—C7—O10	126.3 (2)
O3—N2—C2	119.6 (2)	O9—C7—C7^ii^	117.4 (2)
O5—N3—O6	121.0 (3)	O10—C7—C7^ii^	116.2 (2)
O5—N3—C4	119.9 (2)		

Symmetry codes: (i) −*x*+1, −*y*+1, −*z*+1; (ii) −*x*+1, −*y*+2, −*z*+1.

### Hydrogen-bond geometry (Å, °)

**Table:**

*D*—H···*A*	*D*—H	H···*A*	*D*···*A*	*D*—H···*A*
O1w—H11···O4^iii^	0.84	1.99	2.809 (2)	166
O1w—H12···O5w	0.84	1.89	2.736 (3)	178
O2w—H21···O8^iv^	0.84	2.06	2.890 (2)	174
O2w—H22···O7w	0.84	1.97	2.806 (3)	174
O3w—H31···O7^iv^	0.85	2.07	2.908 (2)	170
O3w—H32···O8w^iv^	0.84	2.03	2.841 (3)	161
O4w—H41···O4^iii^	0.84	1.97	2.759 (2)	155
O4w—H42···O6w^v^	0.83	2.02	2.851 (3)	177
O5w—H51···O6w	0.84	2.06	2.893 (3)	171
O5w—H52···O3^iii^	0.82	2.43	2.956 (3)	123
O6w—H61···O10	0.84	2.09	2.915 (3)	168
O6w—H62···O8w^ii^	0.85	2.13	2.942 (3)	160
O7w—H71···O1^ii^	0.84	1.93	2.766 (2)	172
O7w—H72···O9^vi^	0.84	2.13	2.953 (2)	168
O8w—H81···O6^ii^	0.85	2.46	3.310 (4)	174
O8w—H82···O7w	0.84	2.02	2.816 (3)	160

Symmetry codes: (iii) −*x*+1, −*y*+1, −*z*+2; (iv) −*x*+2, −*y*+1, −*z*+1; (v) *x*, *y*−1, *z*; (ii) −*x*+1, −*y*+2, −*z*+1; (vi) −*x*+2, −*y*+2, −*z*+1.
